# The first 100 days after childbirth: cross-sectional study of maternal clinical events and health needs from primary care

**DOI:** 10.3399/BJGP.2023.0634

**Published:** 2024-08-06

**Authors:** Holly Christina Smith, Patricia Schartau, Sonia Saxena, Irene Petersen

**Affiliations:** Department of Primary Care and Population Health, Institute of Epidemiology & Health, University College London, London.; Department of Primary Care and Population Health, Institute of Epidemiology & Health, University College London, London.; School of Public Health, Imperial College London, London.; Department of Primary Care and Population Health, Institute of Epidemiology & Health, University College London, London.

**Keywords:** general practice, electronic health records, postnatal care, post-partum period, primary health care

## Abstract

**Background:**

The first 100 days after childbirth are important for women recovering from pregnancy and birth.

**Aim:**

To describe the most common clinical events or health needs documented in women’s primary care records in the first 100 days after childbirth.

**Design and setting:**

Cross-sectional study using electronic health records from UK primary care data.

**Method:**

Primary care records were examined from childbirth up to 100 days after childbirth for women aged 16–49 years who had given birth to a single live infant between 2006 and 2016 using IQVIA Medical Research Data. The most common clinical events or health needs based on documented symptoms, diagnoses, and medications were identified. How these varied by patient characteristic was explored.

**Results:**

In total, 925 712 contacts were identified during the 100 days following 309 573 births. Women were most likely to use primary care to have a postnatal visit or check (60.6%, *n* = 187 455), for monitoring (such as a blood pressure reading) (49.9%, *n* = 154 328), and to access contraception (49.7%, *n* = 153 876). Younger women were more likely to have contacts for preventive care compared with older women, but were less likely to have contacts for ongoing mental and physical symptoms or conditions and pre-existing conditions. The highest peak in contacts occurred 42 days after birth, and related to a postnatal check or visit, monitoring a patient, and recording lifestyle factors (such as smoking status).

**Conclusion:**

Primary care services should seek to match the needs of new mothers, taking account of a high volume of contacts, for a broad range of planned and responsive care following childbirth.

## Introduction

Many women are in contact with primary care in the period after they have given birth. Thus, studies estimate that between 47% and 83% of women will report at least one clinical issue around 8 weeks postpartum,^1^^–^3 and women will experience on average 2–6 health needs in the year after childbirth.^4^ Previous studies have found that the most common physical postnatal health needs or symptoms are: fatigue, pain, sex-related concerns, haemorrhoids and constipation, breast problems, and incontinence.^1^^–^4 Although these studies provide some information on the most severe and most common postnatal health needs women experience, the studies typically use self-reported survey methods that may be subject to selection or recall bias, have only included physical health needs, or have limited sample sizes (in the region of 1000).

The use of electronic health records (EHRs) from primary care made it possible, in this study, to add to this literature by using information from clinical practices to explore a broad range of clinical events and health needs from a large cohort of women. In addition, although there is some information about what health needs are likely to be addressed at the 6–8-week maternal postnatal check, no studies, to the authors’ knowledge, have investigated this using EHRs. In the current authors’ previous research, the first 100 days after childbirth was identified as a time of high care for women,^5^ after this, the rate of primary care consultations returns to baseline rates. Thus, in the current study, consultations from the first 100 days after childbirth only were examined as these represent the additional consultations women are having following pregnancy/birth.

The aim of this study was to describe the most common clinical events or health needs documented in women’s primary care EHRs in the first 100 days after childbirth and during their postnatal check.

## Method

### Setting, time period, and data source

Data from one of the largest UK primary care EHR databases, IQVIA Medical Research Data (IMRD), between 2006 and 2016 was used in this study. The database contains patient-level information on: demographics, prescribing, symptoms, procedures, prevention, lifestyle factors, and diagnostics. Diagnostic and symptomatic information are categorised using Read codes, a hierarchical coding system.^6^ Prescribing information is categorised according to the British National Formulary (BNF) classification using BNF codes.^7^ Deprivation information is captured using the Townsend score, which provides a measure of material deprivation based on where a person lives, unemployment, car ownership, home ownership, and household overcrowding. IMRD is broadly representative of the UK population in terms of demographics (age and sex), chronic disease, and mortality; however, there is an over-representation of more affluent people.^8^

**Table:** How this fits in

The first 100 days after childbirth are a crucial time for women as they recover mentally and physically from pregnancy and birth. Previous studies have sought to identify common postnatal conditions and symptoms women may experience after birth, but no studies, to the authors’ knowledge, have used electron ic health records from primary care to exam ine women’s actual care use in this time. The current study found that women most commonly use primary care for: a post na tal check or visit, monitor ing (such as a blood pressure reading), and contra cep tion. The study adds useful knowledge on women’s primary care use following childbirth.

### Participants

The postnatal primary care records of women aged 16–49 years who had given birth to a single live infant recorded in IMRD between 2006 and 2016 were examined. The cohort of women this study draws on has been described in detail previously.^5^

### Variables

#### Primary care contacts

All primary care contacts were included from the date of childbirth up to 100 days after childbirth. A primary care contact was defined as any direct encounter between a patient and healthcare professional taking place in the practice, in a patient’s home, or by telephone, or a prescription issued to a patient. Multiple consultations and/ or prescriptions on the same date were grouped into one contact per woman per day.

#### Categorising health topics in primary care contacts

From these postnatal records, diagnostic, symptomatic information and prescriptions issued were extracted. Diagnostic and symptomatic information is contained within consultations and was categorised using Read codes grouped by their first three digits (Read code stem), and prescription information was detailed using BNF codes grouped by BNF subchapter (for example, 4.3.3). Extracting this information resulted in a long list of codes that were then grouped into distinct categories by adapting established methodology that has been used extensively to create medical and drug code lists,^9^ which then related to the same clinical event or health need. For example, a Read code for postnatal depression, ‘E204.11’, could be grouped with a BNF code for a selective serotonin reuptake inhibitor antidepressant prescription, ‘4.3.3’.

First, key words and known synonyms were identified from the descriptions of the most commonly occurring Read code stems and BNF subchapters. Using the example above, the words ‘depression’ and ‘low mood’ were identified alongside ‘antidepressant prescriptions’. Second, entries that were identified by the authors as relating to the same clinical event or health need were grouped together, in this example they formed ‘depression or low mood’. This process was continued until all Read code stems and all BNF subchapters that occurred >5000 times had been assigned to a group. Other entries were less common and so were not further categorised or reported on. Where entries could fit into >1 group, for example, ‘advice about contraceptives’, a decision was taken to assign these to the most specific group, in this case ‘contraceptives’ rather than ‘education, advice or counselling’. The groupings were initially carried out by one author (the first author) and then cross-checked by all other authors (including two GPs) to ensure reliability and that the groupings made clinical sense. See Supplementary Table S1 for a full list of codes and groups.

These clinical event or health need groups were also further grouped into the following broader categories: acute events or illness; ongoing mental or physical symptoms or conditions (which likely relate to pregnancy or childbirth); pre-existing conditions (conditions that do not directly relate to pregnancy or childbirth); preventive, future health; and other (for example, a type of consultation).

### Statistical methods

The prevalence of the most common clinical event or health needs are given as a rate of the total number of contacts and the total number of women separately. Prevalence rates are given per 100 contacts or per 100 women, and 95% confidence intervals (CIs) were calculated. The analysis was stratified by maternal age, parity, Townsend score, smoking status, calendar year (2-year bands), and mode of delivery. Unadjusted and fully adjusted estimates are presented as prevalence rate ratios (PRRs) and 95% CIs. To account for clustering by GP practice, they were included as a random-effects term. How the contact rate varies across the first 100 days was compared; this is given as a count per 100 contacts on each day and shown as a line chart. In addition, this analysis was restricted to just contacts that took place at the time when the postnatal check was most likely to occur (weeks 5–10 after childbirth).^5^ All analyses were conducted using Stata (version 16).

## Results

### Characteristics

At childbirth, a third of women were aged 30–34 years (31.7%, *n* = 98 269/309 573). There were 21.1% (*n* = 58 583/277 114) in the least deprived Townsend quintile compared with 16.0% (*n* = 44 346) in the most deprived. Three-quarters of women who had a record of delivery method had a vaginal delivery (76.3%, *n* = 75 506/98 932) and the rest had a caesarean birth (23.7%, *n* = 23 426). Over half were a first birth (62.6%, *n* = 149 639/239 107) and 29.0% (*n* = 69 355) were a second birth. Half of women were known to be non-smokers (46.3%, *n* = 143 349/309 573) and 27.6% (*n* = 85 592) as past smokers, which compares with 11.2% (*n* = 34 634) being current smokers (Table 1).

**Table 1.** Characteristics of women at childbirth and number of primary care contacts in the first 100 days after childbirth

**Characteristic**	**All women, *n* (%)**	**Contacts, *n***
**Overall**	309 573	925 712

**Maternal age, years**		
15–19	9568 (3.1)	28 159
20–24	43 116 (13.9)	128 051
25–29	77 698 (25.1)	232 764
30–34	98 269 (31.7)	290 529
35–39	64 171 (20.7)	193 151
40–44	15 908 (5.1)	50 230
45–49	843 (0.3)	2828

**Townsend score, quintile^a^**		
1 (least deprived)	58 583 (21.1)	174 012
2	53 656 (19.4)	159 247
3	62 023 (22.4)	186 314
4	58 506 (21.1)	176 141
5 (most deprived)	44 346 (16.0)	135 566
Missing	32 459	94 432

**Mode of delivery^a^**		
Vaginaldelivery	75 506 (76.3)	255 731
Caesarean	23 426 (23.7)	98 287
Unknown	210 641	571 694

**Parity^a^**		
First	149 639 (62.6)	449 967
Second	69 355 (29.0)	200 600
Third or higher	20 113 (8.4)	60 816
Unknown	70 466	214 329

**Smoking status**		
Currentsmoker	34 634 (11.2)	117 802
Pastsmoker	85 592 (27.6)	262 905
Non-smoker	143 349 (46.3)	426 835
Unknown	45 998 (14.9)	118 170

**Year group**		
2006–2007	63 793 (20.6)	191 326
2008–2009	66 319 (21.4)	201 220
2010–2011	66 478 (21.5)	199 990
2012–2013	63 180 (20.4)	189 546
2014–2015	49 803 (16.1)	143 630

a *Proportions exclude missing or unknown categories.*

### Contacts

For the 309 573 childbirths included in this study; 643 128 consultations and 595 976 prescriptions were identified in the women’s EHRs, resulting in a total of 925 712 contacts of care in the first 100 days after childbirth (multiple consultations and/or prescriptions on the same date were grouped into one contact per women per day). Grouping the most common Read code stems and BNF subchapters resulted in a list of 23 common clinical events or health needs (Table 2). Of all primary care contacts, 81.2% (*n* = 751 439) contained ≥1 of these clinical events or health needs.

**Table 2.** The most common health reasons documented in women’s primary care contacts in the first 100 days after childbirth

**Clinical event or health need**	**Women, *n***	**Rate per 100 women (95% CI)**	**Contacts, *n***	**Rate per 100 contacts (95% CI)**
**Total**	309 573	—	925 712	—

**Acute event or illness**				
Infection	91 766	29.6 (29.5 to 29.8)	135 613	14.6 (14.6 to 14.7)

**Ongoing mental or physical symptoms or conditions**				
Monitoring (for example, blood pressure, pulse rate, and temperature)	154 328	49.9 (49.7 to 50.0)	194 422	21.0 (20.9 to 21.1)
Symptoms or treatments affecting the skin	57 350	18.5 (18.4 to 18.7)	79 173	8.6 (8.5 to 8.6)
Pain (for example, gastrointestinal, backache, or headache)	37 974	12.3 (12.2 to 12.4)	55 594	6.0 (6.0 to 6.1)
Anaemia or blood disorder	28 240	9.1 (9.0 to 9.2)	34 455	3.7 (3.7 to 3.8)
Constipation	22 576	7.3 (7.2 to 7.4)	28 483	3.1 (3.0 to 3.1)
Depression or low mood	22 568	7.3 (7.2 to 7.4)	47 209	5.1 (5.1 to 5.1)
Haemorrhoid or anal abscess	21 818	7.0 (7.0 to 7.1)	26 451	2.9 (2.8 to 2.9)
Breast and breastfeeding related	21 493	6.9 (6.9 to 7.0)	25 597	2.8 (2.7 to 2.8)
Sleep-related, tiredness, or fatigue	1393	0.4 (0.4 to 0.5)	1444	0.2 (0.2 to 0.2)

**Pre-existing conditions**				
Rheumatic disease	27 016	8.7 (8.6 to 8.8)	32 293	3.5 (3.4 to 3.5)
Asthma	13 560	4.4 (4.3 to 4.5)	22 352	2.4 (2.4 to 2.5)
Allergy symptom or treatment	10 265	3.3 (3.3 to 3.4)	12 889	1.4 (1.4 to 1.4)
Thyroid disease or hypothyroidism	6590	2.1 (2.1 to 2.2)	13 369	1.4 (1.4 to 1.5)
Diabetes	6351	2.1 (2.0 to 2.1)	10 195	1.1 (1.1 to 1.1)
Epilepsy	1901	0.6 (0.6 to 0.6)	4499	0.5 (0.5 to 0.5)
Cardiovascular disease	1901	0.6 (0.6 to 0.6)	3630	0.4 (0.4 to 0.4)

**Preventive, future health**				
Contraception	153 876	49.7 (49.5 to 49.9)	183 260	19.8 (19.7 to 19.9)
Lifestyle factors (such as alcohol use, drug use, smoking status, diet, or exercise)	73 538	23.8 (23.6 to 23.9)	80 335	8.7 (8.6 to 8.7)
Education, advice, or counselling	43 373	14.0 (13.9 to 14.1)	51 331	5.5 (5.5 to 5.6)
Cervical examination	27 000	8.7 (8.6 to 8.8)	28 341	3.1 (3.0 to 3.1)
Vaccinations	8522	2.8 (2.7 to 2.8)	9481	1.0 (1.0 to 1.0)

**Other**				
Postnatal check or visit	187 455	60.6 (60.4 to 60.7)	200 769	21.7 (21.6 to 21.8)

### Common postnatal health topics

In the 100 days after childbirth, the most common clinical events or health needs documented in women’s primary care contacts were: a postnatal check or visit (60.6% of women, 95% CI = 60.4 to 60.7, *n* = 187 455/309 573); monitoring (such as a blood pressure reading, or height or weight measurement) (49.9% of women, 95% CI = 49.7 to 50.0, *n* = 154 328); contraception (49.7% of women, 95% CI = 49.5 to 49.9, *n* = 153 876); infection (29.6% of women, 95% CI = 29.5 to 29.8, *n* = 91 766); lifestyle factors (such as alcohol use, drug use, smoking status, diet, or exercise) (23.8% of women, 95% CI = 23.6 to 23.9, *n* = 73 538); symptoms or treatments affecting the skin (18.5% of women, 95% CI = 18.4 to 18.7, *n* = 57 350); and pain (for example, gastrointestinal, backache, or headache) (12.3% of women, 95% CI = 12.2 to 12.4, *n* = 37 974) (Table 2).

### Patterns of primary care contacts

**Figure 1.**
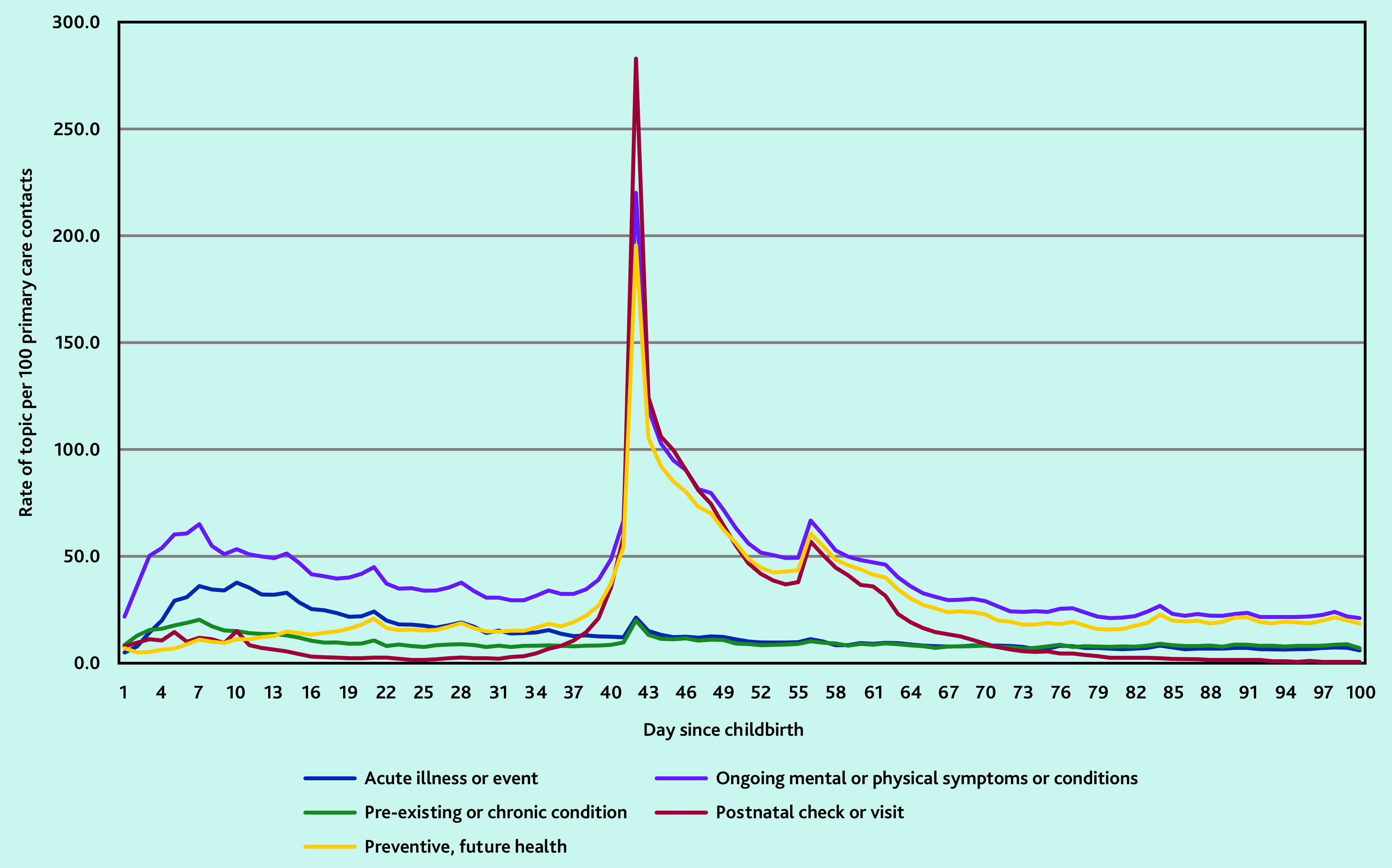
The most common health topic documented in women’s primary care contacts across the first 100 days after childbirth.

There was a small peak in contacts in the first week after childbirth relating to ‘ongoing mental or physical symptoms or conditions’ (65.1/100 contacts) (Figure 1); this peak was largely because of contacts relating to: pain, anaemia or blood disorders, constipation, and haemorrhoid or anal abscess (data not shown). Contacts for ‘acute illness or event’, which includes infections, was also raised across the first 2 weeks after childbirth (peak of 37.8/100 contacts). However, the largest peak in contacts coincided with the postnatal check, on day 42 after childbirth this peaked to 282.9/100 contacts with a record of a postnatal check or visit. There was also a peak in contacts on day 42 for ‘ongoing mental or physical symptoms or conditions’ (221.2/100) and ‘preventive, future health’ (197.3/100).

### Primary care contacts by characteristic

Contacts for pre-existing conditions increased with age and deprivation, and rates of contacts relating to preventive, future health were higher in younger women (see Supplementary Tables S2 and S3). In addition, those who had a caesarean delivery were 39% more likely to have a contact relating to acute events or illnesses (infections) (unadjusted PRR 1.39, 95% CI = 1.36 to 1.44) and 54% more likely to have a contact relating to a pre-existing condition (unadjusted PRR 1.54, 95% CI = 1.49 to 1.59) compared with those with a vaginal delivery (unadjusted PRR 1, reference category) (see Supplementary Table S3). Current smokers were 16% more likely to have a contact relating to a pre-existing condition (unadjusted PRR 1.16, 95% CI = 1.13 to 1.19) and 21% more likely to have a contact relating to preventive, future health (unadjusted PRR 1.21, 95% CI = 1.19 to 1.22) compared with non-smokers. These findings were similar in the fully adjusted models and were broadly similar across other categories and variables, with no major changes over time.

### Common clinical events or health needs at the time of the postnatal check

**Figure 2.**
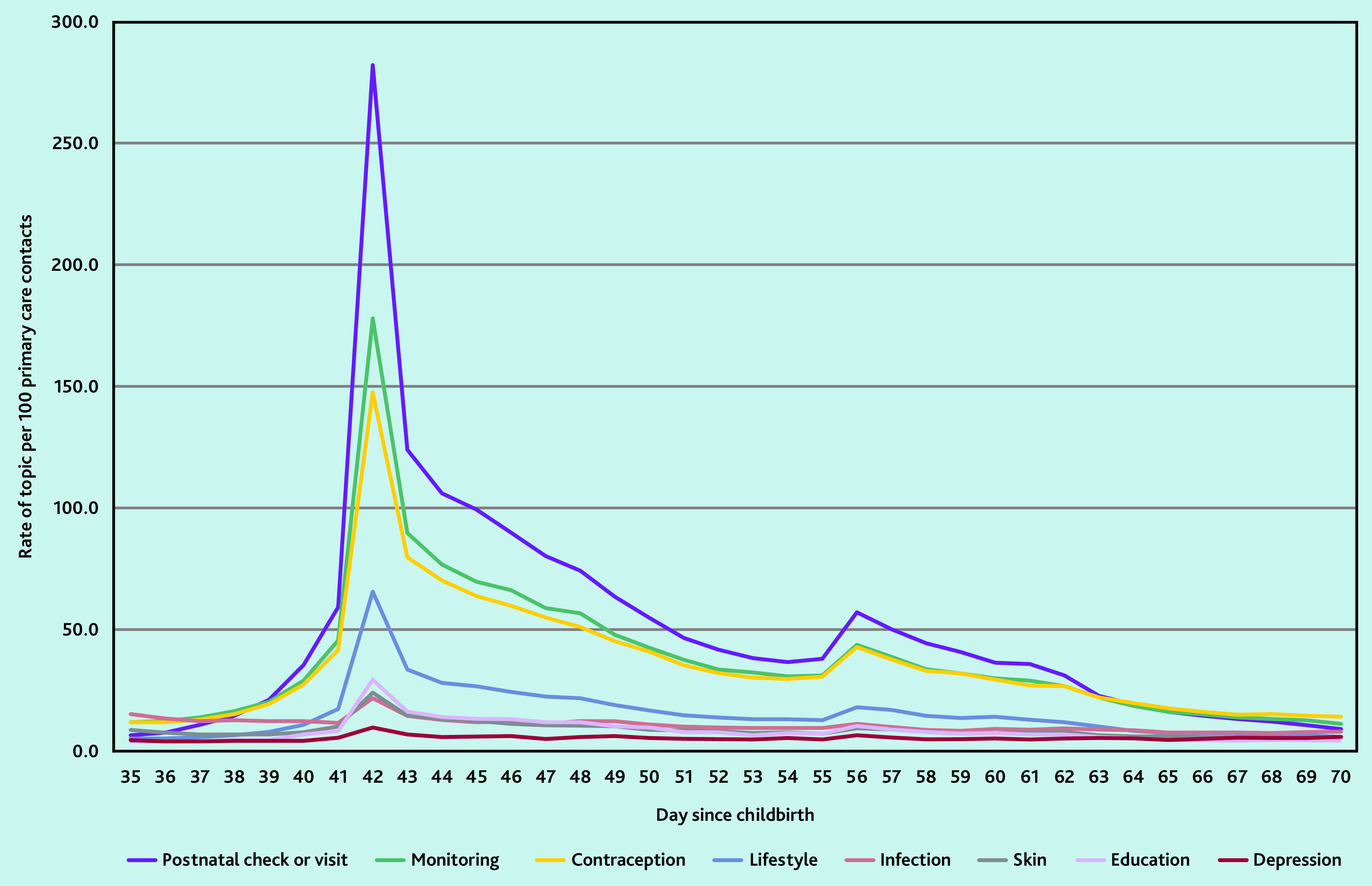
The 10 most common clinical events or health needs documented in women’s primary care contacts between 5 and 10 weeks after childbirth.

Between weeks 5 and 10 (days 35–70) after childbirth, which corresponds to the time of the postnatal check, the most common clinical events or health needs documented were: a record of a ‘postnatal check or visit’; ‘monitoring’ (for example, blood pressure or temperature reading); ‘contraception’; ‘lifestyle factors’ (for example, smoking status); ‘infection’; ‘symptoms or treatments affecting the skin’; ‘education, advice or counselling’; and ‘depression or low mood’ (Figure 2).

## Discussion

### Summary

This study found that in the first 100 days after childbirth, women were most likely to use primary care to have a postnatal visit or check, for monitoring (such as a blood pressure reading, height, or weight measurement), or to access contraception. It was also identified that older women were more likely to have contacts for pre-existing conditions and younger women were more likely to have contacts relating to preventive, future health. In addition, those who had a caesarean delivery were much more likely to have contacts relating to infections (acute events or illnesses). Investigating the peak in consultations at the time of the postnatal check showed that many different health needs are identified and documented at this appointment; most commonly this was for a postnatal check or visit, monitoring, contraception, lifestyle factors, infection, symptoms or treatments affecting the skin, education, advice or counselling, and depression or low mood.

### Strengths and limitations

To the authors’ knowledge, this is the first study to use EHRs to examine why women use primary care after childbirth. Information from 925 712 care contacts relating to 309 573 childbirths was drawn on. The use of EHRs provides a reflection of clinical practice, making it possible to describe a broad range of health topics and clinical events in a large cohort of women. As with all studies of EHRs, however, the authors were limited by what is recorded in the primary care data. Women may experience other needs that are not discussed or documented because they are sensitive, they did not have time to raise them, they sought support from outside primary care, or they did not feel they needed medical help. Thus, this study describes women’s common documented reasons for using primary care services but may lack some elements of women’s broader postnatal health needs and experiences.

Some women may also be more likely to attend primary care services in general, such as those with a pre-existing condition, and therefore may be over-represented in the current study. The authors also acknowledge the limitations in grouping specific Read or prescription codes into clinical events or health needs. Although an approach was taken with cross-checking between authors to ensure reliability, this is still a subjective process, which may lead to some overlap or mis-classification, and other researchers may group these codes differently. However, it is unlikely that changes in these groupings would alter the key findings of this study.

### Comparison with existing literature

Although no previous studies were found examining women’s postnatal primary care use, past studies exploring women’s postnatal health needs provide some useful comparisons. A 2008 integrative review by Cheng and Li summarised studies and found that the most common physical postnatal health needs or symptoms women experience were: pain (20%–79% of women), fatigue or tiredness (15%–76% of women), sore nipples (15%–50% of women), haemorrhoids (36% of women), incontinence (around a third of women), constipation (7%–27% of women), and sex-related concerns (10% of women).^4^ This review did not explicitly look at mental health needs or mastitis. There is some overlap with the current findings as pain (21.9% of women), sleep/ fatigue/ tiredness (1.7% of women), breastfeeding-related needs (9.9% of women), haemorrhoids (9.2% of women), and constipation (9.5% of women) were also identified in the review among the list of common reasons why women use primary care services after childbirth.^4^ The current study typically found lower occurrence of these conditions. For example, just 0.5% of women had a record of experiencing tiredness or fatigue after childbirth and only 7.1% of women in the current study had a record of haemorrhoids. These differences may be because of the differences in data sources used; studies in the review used questionnaires where women self-report conditions or symptoms they experience from a predefined list. It is likely that some of these needs would be more common in a self-reported study, such as tiredness or haemorrhoids, as women experience them but they would not necessarily seek medical intervention and hence not be recorded in primary care data. It is interesting that more sensitive health needs, such as incontinence and sex-related concerns, were not identified in the current study using clinical records but were common in self-report studies. Women may be more reluctant to report these to a healthcare professional or not view these to be medical concerns.

The current study is the first, to the authors’ knowledge, on ‘real-world’ healthcare use after childbirth, thus the authors cannot benchmark clinical events that are not specific conditions and symptoms, such as being monitored or receiving advice, education or counselling, which were identified to be common in this study.

### Implications for research and practice

This study identified numerous health needs documented at the time of women’s postnatal check, including contraceptives, infections, symptoms affecting the skin, and depression and low mood alongside the documenting of valuable clinical events (such as monitoring or lifestyle factors) that demonstrate the varied support provided during these appointments and the value in having a postnatal check. It is also clear, however, that women seek support at other times in the first few months after childbirth and have needs beyond the postnatal check. Particularly for infections, symptoms or treatments affecting the skin, pain, anaemia or blood disorders, constipation, and haemorrhoid or anal abscess, which the current study found to be common in the early days and weeks following childbirth. This has not been identified by previous studies.

Needs may also be different for different groups of women, for example, younger women needing more support for preventive, future health (largely contraception) and those with pre-existing chronic conditions. The importance of supporting these individualised needs and providing personalised care is outlined in NHS England’s Women’s Health Strategy.^10^ Some of these needs may ideally require an earlier appointment (for example, to support those with infections) or longer-term follow-up and additional appointments (for those with chronic health needs, especially in older women). Not all of this needs to be covered during the face-to-face postnatal check with GPs, and women’s preferences in how to access this care may differ. For example, the current results show the need to discuss postnatal contraception, which seems to be mainly taken up by younger women but should really be discussed with all new mothers. As time in the postnatal check is limited, contraception could be addressed with an appointment (virtual or face-to-face) with a member of the nursing team instead. Where appropriate, women could be texted a link, such as to the Contraception Choices website tool (https://www.contraceptionchoices.org), to familiarise themselves with available options to optimise appointment time. Schemes such as the ‘Postnatal contraception in action — north-west London’ scheme, outlined in NHS England’s Women’s Health Strategy,^10^ could be upscaled to ‘free-up’ time to discuss other health concerns in the postnatal appointment.

There may also be benefits in informing women in the first couple of weeks after childbirth about common issues that can arise after giving birth that they may wish to discuss with their primary care team (potentially via letter and/ or text). This could be coupled with an invite for the 6–8-week check and the option to book in for an earlier review should they wish to. In addition, it appears that primary care services may need to engage proactively in identifying more sensitive needs, including incontinence and sex-related concerns. These were commonly identified in self-reported studies but not in the current study using EHRs. Previous research shows that women prefer to be asked about such needs rather than have to offer up this information themselves, and that many women are not routinely asked about this following childbirth despite treatments being beneficial.^11^^,^^12^

Although primary care services may be prepared to offer the planned postnatal check, it is important to see this as part of a pathway rather than ‘the one appointment’. The current findings could also be used to inform a standardised template for the postnatal check to ensure all expected topics are covered and, in particular, to ‘normalise’ topics such as incontinence/ sex-related problems and initiate follow-up for other issues and patient groups (for example, supporting younger women to access contraceptives) that may require longer-term support (for example, those with a chronic condition). Future research should seek to identify what content and delivery of postnatal care is most effective for women.

In conclusion, the first 100 days after childbirth are a crucial time for women as they recover mentally and physically from pregnancy and birth. The study found that in this time women most commonly use primary care for a postnatal check or visit and often monitoring (such as a blood pressure reading) is performed. Likewise, many contacts have records that suggest contraception has been discussed. It was also found that many different health needs are identified at the postnatal check showing the value in having this planned time with women.

## Data

No additional data are available as this work draws on de-identified data provided by patients as a part of their routine primary care. Permissions to use this dataset can be sought directly from IQVIA: https://www.iqvia.com/locations/united-kingdom/solutions/life-sciences-industry-solutions/real-world-solutions/iqvia-medical-research-data
